# Mood Instability Is a Precursor of Relationship and Marital Difficulties: Results from Prospective Data from the British Health and Lifestyle Surveys

**DOI:** 10.3389/fpsyt.2017.00276

**Published:** 2017-12-08

**Authors:** Rudy Cecil Bowen, Lisa Yue Dong, Evyn McMillan Peters, Marilyn Baetz, Lloyd Balbuena

**Affiliations:** ^1^University of Saskatchewan, Saskatoon, SK, Canada

**Keywords:** interpersonal relations, mood, causality, health surveys, self-report

## Abstract

The DSM system implies that affective instability is caused by reactivity to interpersonal events. We used the British Health and Lifestyle Survey that surveyed community residents in 1984 and again in 1991 to study competing hypotheses: that mood instability (MI) leads to interpersonal difficulties or *vice versa*. We analyzed data from 5,352 persons who participated in both waves of the survey. Factor analysis of the Eysenck Personality Inventory neuroticism scale was used to derive a 4-item scale for MI. We used depression measures that were previously derived by factor analyzing the General Health Questionnaire. We tested the competing hypotheses by regressing variables at follow-up against baseline variables. The results showed that MI in 1984 clearly predicted the development of interpersonal problems in 1991. After adjusting for depression, depression becomes the main predictor of spousal difficulties, but MI remains a predictor of interpersonal difficulties with family and friends. Attempts to investigate the reverse hypothesis were ambiguous. The clinical implication is that when MI and interpersonal problems are reported, the MI should be treated first, or at least concurrently.

## Introduction

Criterion 6 in the DSM-5 criteria for borderline personality disorder is a satisfactory description of mood instability (MI), “affective instability due to a marked reactivity of mood (e.g., intense episodic dysphoria, irritability, or anxiety, usually lasting a few hours and only rarely more than a few days)” (1), p. 663. DSM-III described “affect” as “an immediately expressed” and “observed” emotion, contrasted with “mood” which was more “pervasive and sustained” and usually reported (2), p. 353. Because mood implies a feeling state that is more prolonged, we prefer the term mood instability to affective instability because it is known that the trait is pervasive and sustained (3, 4). Prospective environmental momentary assessments depict one mood shifting to a different mood without necessarily passing through a normal phase (1, 5, 6).

The recent DSM editions have been consistent on the theme of mood being reactive to interpersonal stressors. Criteria 1 and 2 of borderline personality disorder describe frantic efforts to avoid abandonment and unstable interpersonal relationships as the core of the syndrome, so it is not surprising that MI is described as evoked by interpersonal difficulties (1). This is one aspect of the theme of identity and interpersonal problems reflecting a borderline organization of the personality. There is support for this concept from the finding that bullying victimization and inadequate parenting interact with dysregulated behavior in the child to predict borderline symptoms (4, 7). In the practice of psychotherapeutic treatment, emotional dysregulation is seen as secondary to interpersonal problems, and the patient is helped to “understand his or her sensitivities and responses” (8).

The term reactive was used by K. Schneider in the 1920s to distinguish depression that was precipitated by a reaction to interpersonal stress as opposed to endogenous depression (9).

He also contrasted people who suffered from vaguely defined ongoing “psychopathy” (i.e., personality disorders) from those with true mental illnesses that affected a person over a definable period of time (9–11). This sets the stage for the DSM-III separation of true psychiatric disorders (axis I) from personality disorders (axis II) such as borderline personality disorder, which were long-lasting, difficult to treat personality aberrations with psychological origins. The personality disorders were predominantly interpersonal and identity-based conditions that were relatively stable, although possibly amenable to long-term psychotherapy (11, 12). When MI is identified clinically, it is usually seen as a diagnostic symptom of a personality disorder (1).

Affective lability characterizes many conditions in DSM-5 including descriptions of affect in children, bipolar mood disorders, and personality disorders but particularly borderline personality disorder. In people with personality disorders, MI is the most common symptom (60%) and MI is present in 80–90% of people with borderline personality disorder (13, 14). However, borderline personality disorder has a median population prevalence of 1.6% (1, 15), and MI is reported in at least 15% of the general population, so mood instability is clearly not confined to people with borderline personality disorder (16, 17).

Recent factor analysis has confirmed Eysenck’s view that MI is a prominent feature of neuroticism (18, 19). In a previous study that factor analyzed the Eysenck Personality Inventory neuroticism scale, a MI factor was found that contains questions such as “mood goes up and down” and “high energy then sluggish” (19). Neuroticism is the most prominent antecedent of anxiety, depression, and suicidal thoughts (20–22) and has immense mental and physical health implications (23, 24). Accordingly, it has been shown that MI as the core feature of neuroticism is closely related to depression, suicidal thoughts, and non-suicidal self-injury (11, 23, 25–27).

Since MI is prevalent in the population, a feature of various disorders, and constitutes a risk for suicidality, our objective in this study was to explore the longitudinal relationship between MI and interpersonal difficulties. The question is whether interpersonal and identity problems predict future MI or is MI the primary problem that predicts subsequent interpersonal difficulties? Resolving the question one way or the other would be important for clinical management when MI and interpersonal difficulties present together in adult patients. Our hypothesis was that MI would predict future interpersonal difficulties.

## Study

### Participants

We used data from the 1984 and 1991 British Health and Lifestyle Survey (HALS) (28). The HALS surveys had the overall objective of studying the relation of various lifestyle factors and overall health. The HALS sample was designed to be representative of the population in England, Scotland, and Wales as measured by the census of 1981. Sampling for the HALS used a three-stage design. 12,672 addresses were sampled randomly from English, Scottish, and Welsh electoral registers. Of these addresses, 96% were verified as home dwellings and from each household a random person 18 years of age or older was interviewed (28). The initial cross-sectional component was completed in 1984 and a follow-up component was completed in 1991–1992. In 1984, there were 9,003 participants (female 5,098) and the mean age was 45.8 years. In 1991, there were 5,352 participants (female 3,052) and the mean age was 51.8 years. Except for the fact that the youngest respondents were now 25 years old, the HALS follow-up sample compares well with the 1991 census data. These data are relevant to the research question because it contains the Eysenck Neuroticism Questionnaire that when factor analyzed yields a prominent MI factor (19) and surveyed participants at two time points. Ethical approval for HALS was received from the British Medical Association ethical committee. As a secondary analysis of anonymized data, the present study was exempted from ethics review.

### Instruments

Health and Lifestyle Survey participants were asked to complete questionnaires for assessing personality and health. These questionnaires were provided by a nurse who asked that these be sent by mail once completed. The first instrument was the 57-item version of the Eysenck Personality Inventory that includes 24 neuroticism items (EPI-N) (Table 1). The 1-month test–retest reliability of the EPI-N scale has been found to be 0.87 (29) and the Kuder–Richardson Formula 20 (KR-20) reliability score in this study is 0.84. The KR-20 is comparable to Cronbach’s alpha for dichotomous item scales. As will be described later in the analysis section, we derived a four-item first factor from the 1984 neuroticism items that reflected MI. These items were “mood goes up and down,” “feel just miserable,” “high energy then sluggish,” and “irritable.”

**Table 1:** Factor loadings of the Eysenck Personality Inventory Neuroticism Scale (1984).

Variable	Factor 1	Factor 2	Factor 3	Factor 4
7. Mood goes up and down	0.63			
9. Feel just miserable	0.52	0.22		
57. Sleeplessness		0.53		
16. Easily hurt	0.23		0.64	
50. Hurt when work criticized			0.66	
26. Highly strung		0.24		0.61
47. Nervous		0.28		0.68
2. Need for friends to cheer up	0.44	0.24		
4. Hard to take no for answer	0.23			
11. Shy with attractive strangers	0.20		0.23	
14. Worry about past actions	0.38	0.24	0.31	
19. High energy then sluggish	0.44			
21. Daydream a lot	0.26			
23. Troubled by guilt	0.30	0.29	0.26	
28. Could have done better				
31. Insomnia	0.23	0.43		
33. Palpitations		0.38		
35. Shaking/trembling		0.34		0.29
38. Irritable	0.34			0.31
40. Worry about awful things	0.28	0.34	0.24	
43. Nightmares		0.34		
45. Aches and pains		0.44		
52. Inferiority	0.21	0.22	0.33	0.23
55. Worry about health		0.42		

In order to investigate the alternative hypothesis that marital problems in 1984 predicted MI in 1991, we calculated a factor score for MI in 1991 to use as a dependent variable in a regression equation. The questions about family/marital problems in 1984 were worded as things about your life now that have a bad effect on your health. Among the choices were “family or marital problems/relationships” and “friends/neighbours/social activity.” Because factor analyzing the 1991 Eysenck Personality Inventory would possibly have yielded a slightly different factor resulting from participant attrition, we summed the same four items: “mood goes up and down,” “feel just miserable,” “high energy then sluggish,” and “irritable” (19).

The 1984 HALS dataset also contained the General Health Questionnaire (GHQ). This is a 30-item questionnaire designed to distinguish people with psychological disturbance from those who are relatively healthy (30). The questions inquire about recent symptoms and changes in ability to carry out daily functions. The GHQ scores correlate well with psychiatric diagnoses (Pearson’s *r* = 0.65–0.7), and sensitivity for psychiatric distress is 80–84% (30). Unlike the Eysenck Personality Inventory, it is not a good measure of lifelong personality characteristics. Goldberg states that the measure is sensitive to affective disorders, and not as sensitive to anxiety disorders (31). The GHQ was appropriate for our purposes because we were interested in a dimensional measure of anxiety/depression, rather than a diagnosis (30). We followed Huppert’s (1989) factor analysis of the HALS’ GHQ and summed the non-overlapping factor A items as a scale for anxiety or mild depression, and corresponding items in factor C as a scale for severe depression (32). The GHQ factor A items (anxiety or mild depression) are: “Felt constantly under strain,” “Found everything getting on top of you,” “Been feeling nervous and strung-up all the time,” “Been feeling unhappy and depressed,” “Been taking things hard,” “Felt you couldn’t overcome your difficulties,” “Lost much sleep over worry,” and “Been losing confidence in yourself” (32). The GHQ factor C items (severe depression) are: “Felt that life isn’t worth living,” “Been thinking of yourself as a worthless person,” “Felt that life is entirely hopeless,” “Been feeling hopeful about your future (reverse),” and “Found at times you couldn’t do anything because your nerves were too bad” (32).

Four variables from the 1991 HALS represented broad areas of interpersonal problems. These were (1) disagreements with spouse/partner in the past year, (2) divorce/separated in the past 7 years, (3) fallen out with a friend/relative in the past year, and (4) lost contact with family/friends in the past year. These were used as the dependent variables in logistic regression models.

## Statistical Analysis

Following our previous procedure, we factor analyzed the 1984 Eysenck Personality Inventory neuroticism subscale to extract a factor that represented MI (19) (Table 2). Factor analysis was conducted using the oblique oblimin rotation because of the correlation that usually exists among psychometric scores (33). We summed “mood goes up and down,” “feel just miserable,” “high energy then sluggish,” and “irritable” items. These are the four items with the highest factor loadings except for “need for friends to cheer up” which was omitted because it contains an obvious interpersonal element that would be related to some of our dependent variables. These four items are also the same as the four items that we isolated previously from the HALS data, but using a slightly different sample (19). Irritability has been shown to be closely associated with MI, as an indicator of major depression (34).

**Table 2:** Logistic regression models with mood instability (MI) score in 1984 as independent variable (adjusted for age and sex).

Independent variables	Dependent variables											
	Divorced during the past 7 years			Disagreement with spouse/partner in the past year			Lost contact with family/friends in past year			Fallen out with friend/relative in past year		
	Odds ratio	Confidence interval		Odds ratio	Confidence interval		Odds ratio	Confidence interval		Odds ratio	Confidence interval	
		2.5%	97.5%		2.5%	97.5%		2.5%	97.5%		2.5%	97.5%
(Intercept)	0.25	0.13	0.49	0.19	0.10	0.35	0.20	0.12	0.34	0.22	0.13	0.37
Age	0.95	0.94	0.96	0.95	0.94	0.96	0.97	0.97	0.98	0.96	0.95	0.97
Sex	1.01	0.75	1.37	1.31	1.00	1.73	0.92	0.73	1.16	1.27	1.00	1.58
MI^a^	**1.15**	1.02	1.29	**1.18**	1.07	1.32	**1.18**	1.08	1.29	**1.25**	1.14	1.37

^a^Sum of the following items: “Mood goes up and down,” “Feel just miserable,” “High energy then sluggish,” “Irritable.”

*Odds ratios that are statistically significant (*p* < 0.05) are in bold*.

To evaluate whether MI explained future relationship problems, we created separate logistic regression models with each of the four interpersonal problems as a dependent variable. In the first set of regression models, MI (1984) was the sole predictor (Table 3). In the second set, MI and anxiety/mild depression (1984) (Huppert factor A) were entered simultaneously (Table 4). In the third set of regression models, MI and severe depression (1984) (Huppert factor C) were entered simultaneously (Table 5). We controlled for age and sex in all analyses.

**Table 3:** Logistic regression models with mood instability (MI) score in 1984 as independent variable (adjusted for age, sex, and anxiety or mild depression).

Independent variables	Dependent variables											
	Divorced in past 7 years			Disagreement with spouse/partner in past year			Lost contact with family/friends in past year			Fallen out with friend/relative in past year		
	Odds ratio	Confidence interval		Odds ratio	Confidence interval		Odds ratio	Confidence interval		Odds ratio	Confidence interval	
		2.5%	97.5%		2.5%	97.5%		2.5%	97.5%		2.5%	97.5%
(Intercept)	0.13	0.06	0.28	0.13	0.06	0.28	0.14	0.08	0.25	0.13	0.07	0.22
Age	0.95	0.94	0.96	0.95	0.94	0.96	0.97	0.97	0.98	0.96	0.95	0.97
Sex	0.97	0.71	1.31	0.97	0.71	1.31	0.90	0.71	1.14	1.22	0.97	1.54
MI^a^	1.02	0.89	1.16	1.02	0.89	1.16	**1.11**	1.00	1.22	**1.12**	1.02	1.24
Anxiety/mild depression^b^	**1.12**	1.07	1.16	**1.12**	1.07	1.16	**1.06**	1.02	1.10	**1.09**	1.06	1.13

^a^Sum of the following items: “Mood goes up and down,” “Feel just miserable,” “High energy then sluggish,” “Irritable.”

^b^Sum of the following items: “Felt constantly under strain,” “Found everything getting on top of you,” “Been feeling nervous and strung-up all the time,” “Been feeling unhappy and depressed,” “Been taking things hard,” “Felt you couldn’t overcome your difficulties,” “Lost much sleep over worry,” “Been losing confidence in yourself.”

*Odds ratios that are statistically significant (*p* < 0.05) are in bold*.

**Table 4:** Logistic regression models with mood instability (MI) score in 1984 as independent variable (adjusted for age, sex, and anxiety or severe depression).

Independent variables	Dependent variables											
	Divorced during the past 7 years			Disagreement with spouse/partner in the past year			Lost contact with family/friends in past year			Fallen out with friend/relative in past year		
	Odds ratio	Confidence interval		Odds ratio	Confidence interval		Odds ratio	Confidence interval		Odds ratio	Confidence interval	
Variables		2.5%	97.5%		2.5%	97.5%		2.5%	97.5%		2.5%	97.5%
(Intercept)	0.13	0.06	0.29	0.09	0.04	0.18	0.18	0.09	0.35	0.10	0.05	0.19
Age	0.95	0.94	0.96	0.95	0.94	0.96	0.97	0.97	0.98	0.96	0.95	0.97
Sex	0.97	0.71	1.31	1.26	0.96	1.66	0.93	0.74	1.18	1.20	0.95	1.51
MI^a^	1.07	0.94	1.21	**1.13**	1.01	1.26	**1.16**	1.05	1.28	**1.18**	1.07	1.30
Severe depression^b^	**1.10**	1.03	1.16	**1.10**	1.04	1.16	1.01	0.96	1.07	**1.10**	1.05	1.15

^a^Sum of the following items: “Mood goes up and down,” “Feel just miserable,” “High energy then sluggish,” “Irritable.”

^b^Sum of the following items: “Felt that life isn’t worth living,” “Been thinking of yourself as a worthless person,” “Felt that life is entirely hopeless,” “Been feeling hopeful about your future (reverse),” “Found at times you couldn’t do anything because your nerves were too bad.”

*Odds ratios that are statistically significant (*p* < 0.05) are in bold*.

**Table 5:** Linear regression model of family or marital problems (1984) as independent variable on mood instability (1991).

Variables	Estimate	SE	*t*	*p* (>|*t*|)
(Intercept)	5.80	0.15	39.51	0.000
Age	0.02	0.00	12.34	0.000
Sex	−0.29	0.04	−7.01	0.000
Family/marital problem (1984)^a^	0.02	0.01	1.35	0.178

^a^The participant responded “Yes” to the question “Family or marital problems/relationships had a bad effect on health.”

In an attempt to address the causal direction, we compared a regression model in which MI predicted future relationship problems as described above, with one in which relationship problems in 1984 predicted MI in 1991. The questions in 1984 were not the same as they were in 1991. The question was “are there things about your life now that has a bad effect on your health?” Among the options were “family or marital problems/relationships” and “friends/neighbours/social activities.” We reasoned that if the association was statistically significant in one direction and not significant in the other, then the model showing an association probably reflected the correct causal direction.

R and RStudio. version (0.98.1103) (35) were used for the statistical analyses.

## Results

Disagreements with the spouse/partner (prevalence: 6%) were slightly more common than divorce and separation (prevalence: 5%). Falling out with family/friends was the most common interpersonal problem (prevalence 10%), while the problems with families and friends (9–10%) were generally more common than the problems with spouse/partner (5–6%). These are consistent with what might be expected.

Table 1 summarizes the results of the factor analysis of the Eysenck Personality Neuroticism Scale. We examined the eigenvalues and the scree plot and concluded that four factors needed to be retained. The first factor represents MI with the highest loading for “mood goes up and down” (loading: 0.63) and the second highest loading being “feel just miserable for no reason” (loading: 0.52).

As Table 2 shows, MI in 1984 predicts all 4 social problems 7 years later (*p* < 0.05). When anxiety/mild depression (Table 4) and then severe depression (Table 5) were added to the regression model, for “lost contact with family/friends in past year” and “fallen out with friend/relative in past year,” MI, mild and severe depression were all statistically significant (except that severe depression is not significant for “lost contact with family/friends in past year”). For the outcomes “divorced during the past 7 years” and “disagreement with spouse/partner in the past year,” MI is no longer a significant predictor while depression was significant (except for “disagreement with spouse/partner in the past year” where both MI and severe depression are both statistically significant).

The Pearson correlation between mild and severe depression (omitting the overlapping items) is 0.992 indicating that they essentially represent the same construct, and they performed similarly in the regression models in Tables 2 and 3. Measurements were made at only two time points (1984 and 1991), so we cannot comment on the time course of depression, but we obtained equivalent results whether 1984 or 1991 depression was entered into the regression models.

Tables 5 and 6 shows the results of the investigation of the alternate hypothesis that belief that family/marital problems/relationships or friends/neighbors/social activity has a bad effect on your life in 1984 predicts MI in 1991 (β = 0.017, *p* = 0.178). Family/marital problems in 1984 did not predict MI in 1991. Problems with friends/neighbors/social activity did not have a significant effect (β = 0.050, *p* = 0.0621).

**Table 6:** Linear regression model of friend, neighbors/social problems (1984) as independent variable on mood instability (1991).

Variables	Estimate	SE	*t*	*p* (>|*t*|)
(Intercept)	5.50	0.26	21.21	0.000
Age	0.02	0.00	12.31	0.000
Sex	−0.29	0.04	−7.13	0.000
Friend/neighbors/social problems (1984)^a^	0.05	0.03	1.87	0.06

^a^The participant responded “Yes” to the question “Friends/neighbors/social activity had a bad effect on health.”

## Discussion

The results of this study indicate that MI precedes and predicts the development of disagreements with the partner/spouse and divorce/separation, and also disagreements with relatives and friends. It has been shown that MI in the context of relationship difficulties eventually leads to the development of more pervasive low mood or depression (25). When depression develops, the results indicate that MI along with depression continues to be a significant determinant of disagreement with friends and relatives. For disagreements with the partner/spouse and divorce, our results show that depression becomes the sole predictor (with one exception).

Mood instability by definition suggests frequent and sudden changes in low and high moods, and also anxiety and irritability (1). The switches in mood can occur for no apparent reason and can appear as abrupt and unpredictable (36). Family and friends usually reside in separate abodes from adults with MI, and people with MI can postpone communication and socialization until their mood is more positive. This means that family and friends are not as likely to be exposed to the full effects of continued pervasive depression compared to spouses who would be continually exposed. Family and friends might notice fluctuating inconsistency, but this may not be as detrimental to relationships as the more pervasive pessimism of depression and irritability.

Other studies have reported that affective instability predicts romantic impairment (24, 37, 38). The paper by Lahey (24) gives an excellent discussion of the association between marital distress and neuroticism. Among married people, irritability is usually directed primarily at the partner/spouse, who is most accessible (37–40). This can be distressing to the partner or spouse. In addition, accompanying anxiety is often associated with limitations in activities, including social activities (41). Anxiety can also lead to dependence on the partner/spouse, which is often resented and can precipitate reciprocal rejection (42). It is likely that the person with MI will have less personal support, and is likely to be at risk for a variety of physical complaints and illnesses (24). There is substantial evidence that neuroticism in either partner is associated with lower satisfaction with the relationship and higher divorce rates (43). It is worth noting that the neuroticism (N) scale in the NEO personality inventory consists of questions about negative emotions, such as sadness and anger, and it is an advantage of the EPI neuroticism scale that it also includes questions about MI (19, 43, 44).

We attempted to explore the alternate hypothesis that marital difficulties in 1984 predicted MI in 1991. Unfortunately, the questions in 1984 about relationship difficulties were worded differently from the 1991 questions and so the results are ambiguous. The results suggest that family or marital problems reported as having a negative effect on one’s health do not give rise to unstable moods in the future. This is further discussed in the limitations section.

There is prospective evidence that childhood abuse can have an effect on the development of mood and personality problems, including affective instability that resembles borderline symptoms in adults (24, 45–47). There is also evidence that negative emotionality and behavior problems in children challenge the parents’ repertoire of skills resulting in worsening symptoms in the adolescent (4, 20, 45). It has been shown that dysregulated behavior may challenge peer relationships resulting in bullying which worsens symptoms in the subject (7). Some of this work is prospective, overcoming the main problem of retrospective reports (4, 45). It is also known that people with emotional difficulties, in the absence of clear causes, may attribute the emotional difficulties to immediate recent identifiable events, such as interpersonal problems (48). Studies are needed on the genetic origins of MI as has been done for neuroticism (23, 24, 49).

There are limitations to this study. The data are not recent but we were interested in relationships between variables which are not likely to change over a few decades, as opposed to prevalence. The measures of interpersonal difficulties were single items but addressed relevant interpersonal problems. Four measures were available, and the results were consistent. There are many reasons for relationship problems with a spouse or family or friends and the study addressed just two of these, MI and depression. The study also did not account for assortative mating of couples (50). Unfortunately, the questions about interpersonal problems in 1984 were not identical to those used in 1991. Participants were asked “Are there things about your life now that have a bad effect on your health?” The question then further specified if it occurred from either (1) family or marital problems/relationships, or (2) friends/neighbors/social activities. Since reporting relationship problems were contingent on having a bad effect on health, it is possible that relationship problems *per se* lead to MI. This would be the case if relational problems were not severe enough to have an effect on health or if they were, that the participant responded “No” for whatever reason.

That MI precedes relationship problems is clinically relevant because patients frequently report experiencing both types of difficulties (8). Two-year follow-up of patients with borderline personality disorder found that mood symptoms and interpersonal difficulties tend to improve with focused psychological treatment, although most patients are still in the symptomatic range at follow-up (51). Symptoms tend to remit and recur, and the mood symptoms are particularly resistant to change (14). When they present together, the results suggest that the MI should be treated as the primary problem and the relationship difficulties as secondary to the MI. This is contrary to the public perception that relationship difficulties are one of the main causes of MI and subsequent depression, although there is evidence that public attitudes are changing toward a more biogenetic view, particularly among the young (52, 53). This approach to treatment could conceivably reduce the rate of marital difficulties and separation. Depression with MI tends to be treated with antidepressants for which there is little evidence of effectiveness (8, 54–56). Theoretically, mood stabilizers should be studied more extensively, but unfortunately the data are currently thin (8, 55).

## Conclusion

Mood instability predicts subsequent marital problems and divorce, and disagreements with relatives and friends in adults. There is less evidence that interpersonal problems predict later MI. This has implications for the sequencing of treatment of MI and marital and interpersonal problems. Treating MI first is likely to result in greater improvement, but this needs to be tested empirically.

## Ethics Statement

As a secondary analysis of anonymized data, the study was exempted from ethics review. HALS received approval from the British Medical Association ethical committee.

## Author Contributions

RB conceptualized the study, and wrote the initial and final drafts. LD performed the statistical analysis, and wrote the methods and results of the initial draft. EP helped interpret the results and critically revised the manuscript. MB critically reviewed the results and contributed to the discussion. LB assisted in the statistical analysis and rewrote the initial draft. All authors reviewed and approved the submission.

## Conflict of Interest Statement

The authors declare that the research was conducted in the absence of any commercial or financial relationships that could be construed as a potential conflict of interest.
